# Walking on the tightrope: the shared roles of the bridging pericytes in the brain

**DOI:** 10.3389/fncel.2025.1615579

**Published:** 2025-07-22

**Authors:** Audrey Chagnot, Axel Montagne

**Affiliations:** ^1^UK Dementia Research Institute, The University of Edinburgh, Edinburgh, United Kingdom; ^2^British Heart Foundation - UK Dementia Research Institute Centre for Vascular Dementia Research, The University of Edinburgh, Edinburgh, United Kingdom; ^3^Centre for Clinical Brain Sciences, The University of Edinburgh, Edinburgh, United Kingdom

**Keywords:** pericytes, brain, capillaries, bridging cells, tunneling nanotubes, pericyte migration, vascular regression

## Abstract

The vasculature of the central nervous system (CNS) is a highly specialized structure that delivers oxygen and nutrients to energy-demanding neural cells while protecting them from the toxicity of blood-borne substances. Pericytes, located alongside microvessels, coordinate with endothelial cells to maintain the integrity of the blood-CNS barriers and to regulate vascular responses to neural activity. Pericytes extend processes that typically wrap around or align the endothelial cells, remaining embedded within the vascular basement membrane. Occasionally, however, some of these processes detach and form bridges between separate capillaries. These bridging structures are the focus of ongoing debate. While some studies propose they serve as tunneling nanotubes mediating neurovascular coupling, others argue they may be remnants of vascular regression or involved in the process of pericyte migration. In this review, we aim to clarify these varying interpretations of bridging pericyte processes and provide a unified understanding to guide future research. We discuss their reported roles in both CNS health and disease, highlighting their potential significance in vascular aging and rejuvenation.

## 1 Introduction

### 1.1 The elusive definition of pericytes

Pericytes are mural cells found along microvessels, lining the abluminal side of the endothelium, and are present in all vascularized tissues of the body. The earliest references to cells wrapping around capillaries, characterized by contractile activity and a nucleus bulging on the outside, can be traced back to independent publications by Rouget and Eberth in the late 19th century (Eberth, 1871; Rouget, 1874). These early descriptions gave rise to the now-classic “bump-on-a-log” pericyte morphotype. This foundational work was greatly expanded 50 years later by the German anatomist Zimmermann, whose landmark paper (Zimmermann, 1923) offered a comprehensive characterization of these cells.

In his extensive study, Zimmermann observed silver-stained cells lining capillaries, venules, and arterioles across various vertebrate tissues, which he named “pericytes” (*Pericyten*). Unlike smooth muscle cells (SMCs) found on larger vessels, pericytes present a range of morphologies related to their position, or zonation, along the vascular tree, whether on pre-capillary arterioles, capillaries, or post-capillary venules (Zimmermann, 1923).

As might be expected for cells described as existing along a continuum rather than fitting into a fixed category, the definition of pericytes has fluctuated over time. While multiple pericyte markers have been used, such as CD13 (Aminopeptidase N) (Kunz et al., 1994), NG2 (Neural/glial antigen 2), PDGFRβ (Platelet-derived growth factor beta) (Franklin et al., 1981), and Atp13a5 (ATPase 13a5) (Guo et al., 2024), they may also be expressed by other cells like SMCs and fibroblasts. As a result, pericytes are often defined using a combination of zonation, morphology, and marker expression (Attwell et al., 2016; Krueger and Bechmann, 2010).

These inconsistencies in definition become particularly important when they influence conclusions about the functional roles and pathophysiological relevance of pericytes. One such point of contention is their contractile capacity, which has long been, and remains, debated (Attwell et al., 2016; Hall et al., 2014; Hartmann et al., 2022, 2021; Hill et al., 2015; Hørlyck et al., 2021; Krueger and Bechmann, 2010). While we will not delve further into this debate in the present review, it is worth noting that, for example, the conflicting view on pericyte expression of actin, a part of the contractile machinery, may stem from experimental differences (Alarcon-Martinez et al., 2018; Bandopadhyay et al., 2001; Nehls and Drenckhahn, 1991).

### 1.2 Pericytes and endothelial cells—an intimate story

Traits that are unambiguously shared by all pericytes and which are crucial part of their functional roles are their embedding within the vascular wall and their close association with endothelial cells (Krueger and Bechmann, 2010). This intimate relationship can be observed in the so-called “peg-and-socket” junctions (Leeson, 1979), interdigitations of the plasma membranes of the two cell types, which not only reinforce mechanical attachment but increase contact surface area. These structures are mostly located beneath the pericyte soma but can also be found along the borders of pericyte processes (Abdelazim et al., 2022; Ornelas et al., 2021).

Further reinforcing this tight association, numerous junctional complexes, such as gap and dense/cadherin junctions, interlink pericytes and endothelial cells (Sims, 1986). While pericyte-to-pericyte junctions do exist, endothelial cells remain their primary coupling partners (Kovacs-Oller et al., 2020). This critical cellular axis appears early during development (Payne et al., 2021) and plays key roles in both neurovascular coupling (Grubb et al., 2021) and endothelial stabilization (Ayloo et al., 2022; Park et al., 2017).

Because of this close interaction, pericyte coverage of the endothelium is often used as an indicator of microvascular health. A reduction in pericyte coverage has been associated with aging (Berthiaume et al., 2022), diabetes mellitus (Corliss et al., 2020; Pfister et al., 2008), glaucoma (Alarcon-Martinez et al., 2022), hypertension (Baggeroer et al., 2024), inflammation (Medina-Flores et al., 2023) and various neurovascular diseases, including Alzheimer’s disease (Ding et al., 2020; Halliday et al., 2016; Montagne et al., 2015).

However, when comparing pericyte coverage across studies, methodological differences must be considered. In most electron microscopy (EM)-based studies, pericyte coverage is quantified as the ratio of the pericyte-to-endothelial cell surface contact relative to the total external surface of the endothelial cell. Compared to immunofluorescence-based methods, the use of ultrathin sections in EM allows for a more precise measurement of this contact. However, due to EM’s inherently limited field of view, such analyzes are generally confined to a small number of vessels. In contrast, immunofluorescence studies typically estimate coverage by calculating the area of pericyte marker expression relative to that of vascular markers. While this latter approach allows for quick estimations, it has notable limitations, particularly the loss of spatial information from analyzing Z-projections of 3D stacks. To improve reproducibility, we developed a FIJI macro code that provides users greater control over pericyte coverage measurements (Chagnot and Montagne, 2024; Munro et al., 2024).

Reported pericyte-to-endothelial cell ratios depict the CNS as one of the richest organs, with often cited values ranging from 1:1 in retina to 1:3 in the cortex. In contrast, ratios in the lung and skeletal muscle are commonly cited as low as 1:10 and 1:100, respectively. However, it is worth noting that these extreme figures arose from isolated observations rather than aggregated data (Frank et al., 1987; Shepro and Morel, 1993), and may even be inaccurate, as some studies suggest that the number of pericytes in muscle are comparable to, or even exceed, those in the retina (Tilton et al., 1985; Williamson et al., 1980). Advanced methodologies pairing immunohistochemistry with single-cell omics allow more precise evaluations of cell numbers in tissues (Mäe et al., 2021).

### 1.3 Pericyte subtypes—a question of processes?

**FIGURE 1:**
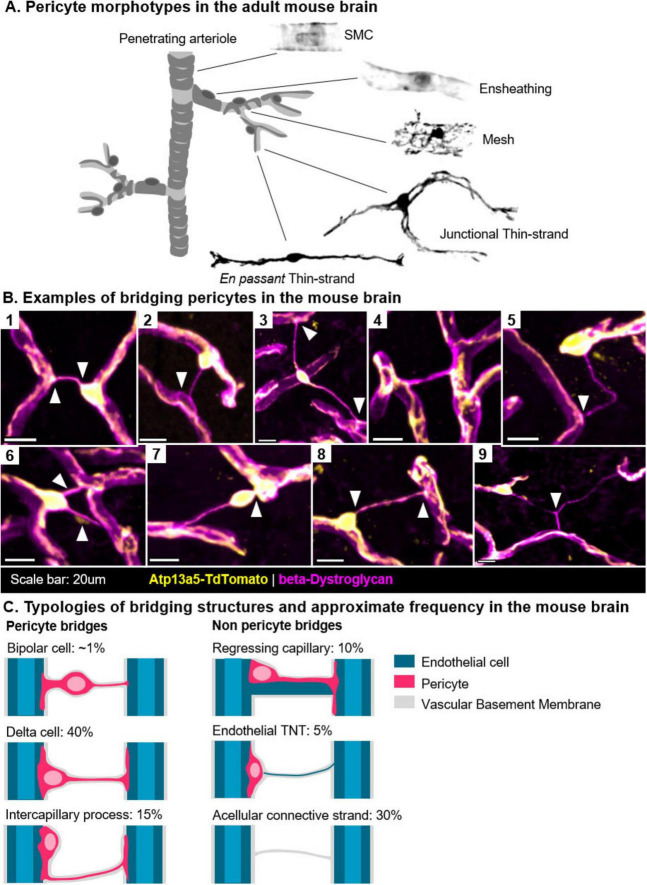
Bridging pericytes in the adult mouse brain. **(A)** Pericytes exhibit a variety of morphologies that reflect their zonation along the vascular tree, here presented by Atp13a5 + cells. In contrast, smooth muscle cells (SMCs) are primarily contractile, lack an ovoid soma, and are located along the penetrating arterioles. **(B)** Visualization of bridging pericytes in the mouse brain (Atp13a5: yellow, β-Dystroglycan: magenta). (B1) A classic “delta” morphology, with an endfoot extending to a neighboring capillary (arrow). (B2) Interaction between an Atp13a5 + cell and an Atp13a5- cell, potentially representing a pericyte-to-endothelial tunneling nanotube (TNT). (B3) A bipolar cell located midway along a long bridge. (B4) A bridge containing an Atp13a5 + process, though distant from the originating soma. (B5) A tortuous Atp13a5- bridge connected to a cell soma. (B6) A rare occurrence showing two bridges emerging from a single soma. (B7) A bipolar cell with a thickened process anchoring it to the nearest capillary. (B8) A delta-shaped cell. (B9) A rare occurrence of a Y-shaped bridge, with a delta cell positioned at its left extremity. **(C)** Summary of the different bridge morphologies observed in the adult (6-month-old) mouse brain. In over half of the bridges, pericytes were the dominant cellular component. In approximately one-third of cases, cellular processes could not be clearly identified.

Most of the morphological diversity observed in pericytes is related to their processes (Figure 1A). As early as 1923, Zimmerman described primary processes, which emanate directly from the cell’s ovoid soma and extending longitudinally along the vessel, and secondary processes, which enwrap the underlying capillary (Zimmermann, 1923).

Zonation along the vascular tree has been proposed as a way to identify pericyte subtypes (Grant et al., 2019). Branch order 0 starts at the penetrating arteriole, increasing with each new branch. Pericytes are considered absent from penetrating arterioles and larger vessels, where they are replaced by SMCs. While this classification is convenient when the entire vascular tree is accessible for annotation, it may not be the case in thin immunohistochemistry or electron microscopy sections. Advances in omics technologies offer promising avenues for exploring the vascular landscape; however, translating these molecular insights into conventional pericyte morphologies remains an open challenge (Sewell et al., 2025).

Pericytes found on precapillary arterioles (branch orders 1–4) are referred to as “ensheathing pericytes” due to their formation of a continuous, uniform sleeve completely wrapping the arteriole (Hartmann et al., 2015; Grant et al., 2019). The identification of these cells as *bona fide* pericytes has been contested, given their similarities to SMCs located upstream on arterioles and arteries (Attwell et al., 2016; Hill et al., 2015). Their contractile nature and expression of α-SMA (alpha smooth muscle actin) are not debated. However, their protruding ovoid cell body, absent in SMCs, has led some researchers to classify them as pericytes in keeping with Zimmermann’s historical definition (Grant et al., 2019). While the distinct morphology of ensheathing pericyte is almost certainly a consequence of differential gene expression relative to SMCs, no molecular marker has yet been identified that reliably discriminates between these two cell types.

At the junction between penetrating arterioles and their downstream branches, contractile “precapillary sphincters” have been described. These are formed and supported by ensheathing pericytes. By reducing the lumen size at this interface, these sphincters help to reduce the pressure load onto the downstream microvessels (Grubb et al., 2020).

Capillaries of the lowest order, those closest to arterioles, are typically covered by “mesh pericytes”, which extend a network of apparently disorganized processes over their vascular territory, creating a mesh-like appearance (Hartmann et al., 2015; Grant et al., 2019). These cells are thoughts to represent the transition toward the most common pericyte morphotype: the “thin-strand pericyte”.

Thin-strand pericytes are predominant along the capillary bed. They are characterized by long, thin primary processes that run along the vessel. Often spanning multiple capillary branches, their soma are frequently located at vascular bifurcations (Hartmann et al., 2015; Berthiaume et al., 2018). Tiny secondary processes extend tangentially and regularly from the primary ones, giving them a caterpillar-like appearance in electronic microscopy (Sims, 1986). These processes are dynamic, especially at their terminal segments, and can rapidly expand to cover exposed endothelial cells following the loss of a neighboring pericyte (Berthiaume et al., 2018). Subclassifications have also been proposed, such as “junctional pericytes”, with bodies at bifurcations, and “*en passant* (passing by) pericytes”, located along capillaries (Ornelas et al., 2021).

Post-capillary venules are also covered by mesh pericytes morphologically similar to those found upstream. These cells appear to represent a transitional type toward venular SMCs, which adopt a more stellate morphology at this level (Smyth et al., 2018).

Given the intimate interactions between pericytes and endothelial cells, the existence of pericyte processes and structures detached from the endothelium raises question about their nature and function. In the following section, we will explore the historical background of these so-called bridging cells and the various ways they have been described in the literature.

## 2 Bridging cells in the central nervous system

### 2.1 Early reports: comparative anatomy and structural characterization

In his seminal 1923 paper, Zimmermann described various “pericytes” morphologies on capillaries across a wide range of vertebrate tissues. He noted that these cells occasionally extend primary processes between neighboring vessels, a phenomenon he observed in all examined tissues. While he hypothesized that these extensions might transmit signals between capillaries, he also speculated that they could originate from regressing endothelial cells (Zimmermann, 1923).

Forty years later, Cammermeyer introduced the term “delta cells” to describe triangular-shaped somas found at the base of intervascular strands in the CNS. Occasionally, he observed somas located midway along the strands (Figure 1B). He described a variety of shapes including Y-shaped structures and noted that these formations were most prevalent in the cerebellum, midbrain, and medulla, while frontal regions such as the cortex contained relatively few. Across species, birds exhibited significantly more bridges than mammals, a difference he attributed to a structural function of the bridges, suggesting they might help organize and stabilize capillary loops. He speculated that in a context of edema, these bridges might become deleterious, compressing capillaries and causing ischemia (Cammermeyer, 1960).

In a follow-up study, Cammermeyer reported the presence of “granules” and “vacuoles” within these bridges, proposing they might serve as routes for the transmission of signals and substances between vascular territories. He also described an orchestrated presence of various cell types around the bridges, including microglia, oligodendrocytes, and, in the peripheral nervous system, mast cells (Cammermeyer, 1965).

As the morphological diversity of these “bridges”, “intervascular strands”, and “string capillaries” became apparent, Guseo and Gallyas proposed a classification based on brightfield observations of silver-stained human brains. They distinguished three types: (1) endothelial protrusion giving rise to new capillaries, (2) collapsed or regressing capillaries, and (3) pericyte-based structures. Type (1) was common in newborn brains, particularly in myelinating areas, while types (2) and (3) were more prevalent in adult and pathological tissues (Guseo and Gallyas, 1974).

As originally reported by Zimmermann and Cammermeyer, these bridges are not exclusive to the CNS (Cammermeyer, 1965, 1960; Zimmermann, 1923). Similar structures were observed in the skin (Imayama and Urabe, 1984) and muscle (Williamson et al., 1980), typically interpreted as playing structural roles.

In 1988, Leibnitz and Bär published a comprehensive report on “bridging cells in the brain”, proposing that they represent a subtype of pericytes. They highlighted the inherent asymmetry of these structures, with a spindle or bell-shaped cell, with a process ending on a neighboring capillary in a disc- or pyramid-shaped thickening. Even “bipolar cells” located within strands displayed a process significantly thicker than the other. They also observed that apparent bending of capillaries near bridges, which they interpreted either as mechanical tension or as endothelial cell proliferation. Though cautious in their conclusions, they proposed that bridges could represent pericyte detachment as well as serve as guides for angiogenesis. Nonetheless, they argued that the relative rarity of these structures made them unlikely contributors to neurovascular coupling (Leibnitz and Bär, 1988).

### 2.2 Bridging cells as migrating pericytes

In the retina, bridging cells are often observed in the context of diabetes mellitus. Experimental hyperglycemia is sufficient to trigger pericyte detachment and migration from capillaries via pathways involving angiotensin-2 and PDGFRβ. This process can be rescued with insulin or the PDGFRβ inhibitor imatinib (Corliss et al., 2020; Pfister et al., 2008). Similar phenomena have been observed in the cochlea following acoustic trauma (Hou et al., 2018). In these settings, bridges are interpreted as hallmarks of a pathological process marked by pericyte loss and microvascular destabilization.

### 2.3 Bridges as tunneling nanotubes

Intercellular communications through protrusions of the plasma membrane were first reported 20 years ago by Rustom et al. (2004). These so-called “tunneling nanotubes” or TNTs were originally observed in cultured cells and defined by their sub-micron diameters and considerable lengths, often exceeding the size of the originating cell. Their lifecycle ranged from a few minutes to several hours and featured an actin-rich, tubulin-poor cytoskeleton. However, as TNT-like structures were reported across multiple models, it became apparent that a continuum of superficially similar structures exists (Korenkova et al., 2020), often termed cytoplasmic bridges (Haglund et al., 2011) or cytonemes (Sowinski et al., 2011). TNTs form in virtually all animal tissues and are diverse in nature and functions, encompassing bundled actin/tubulin hybrids (Sartori-Rupp et al., 2019) and involved in organelle transfer (Önfelt et al., 2006), electrical coupling (Wang et al., 2010), and even in the spread of misfolded proteins in neurodegenerative disorders (Zhang et al., 2021; Zhang, 2011).

A recent interpretation suggests that the bridges observed in the CNS may be TNTs extending between pericytes. Alarcon-Martinez et al. (2020) described these inter-pericytes TNTs in the mouse retina as exhibiting hybrid characteristics, sharing features of both pericyte processes and the TNTs originally described by Alarcon-Martinez et al. (2020), Rustom et al. (2004), Zurzolo (2021). Their study demonstrated mitochondrial transport through these structures (Alarcon-Martinez et al., 2020), reminiscent of the “granules” observed by Cammermeyer (1965), a phenomenon also reported in some TNTs (Kempf et al., 2024; Wang and Gerdes, 2015). However, unlike classical TNTs that connect two distinct cells, these bridges appear more akin to extensions of a singles pericyte. Specifically, Alarcon-Martinez et al. (2020) showed that the “TNTs” they identified established cytoplasmic continuity between two parts of the same pericyte spanning different capillaries. This was confirmed through electroporation of a high molecular weight tracer. In their study, bridging pericytes were described as comprising a soma containing the nucleus and most of cell mass, an “endfoot” in contact with another capillary, and a connecting structure, the so-called “TNT”. Rather than linking two separate cells, as bona fide TNTs do, these bridges resemble elongated pericyte processes that span adjacent capillaries, consistent with early observations by Zimmermann (1923).

A more surprising connection made in the same study suggests a potential role for pericyte bridges in neurovascular coupling. Using a Ca^2+^ activity reporter, the authors found synchronized calcium transients between pericytes connected by bridges, with opposite capillary diameter changes upon light stimulation. Bridge disruption during ischemia led to desynchronization (Alarcon-Martinez et al., 2020). Follow-up work showed that glaucoma-induced elevations in intracellular calcium also led to bridges ruptures (Alarcon-Martinez et al., 2022). It is plausible that the nature of communication occurring along bridging structures resembles that of pericyte processes, although this has yet to be confirmed. The opposing coupling response reported by Alarcon-Martinez et al. (2020) 2022 may represent a further refinement of the neurovascular response, but the underlying mechanisms remain to be elucidated. One possibility is that contraction of the pericyte bridge results in a form of capillary constriction or “strangulation”, as originally proposed by Guseo and Gallyas (1974).

While the bridges presented in the works of Alarcon-Martinez et al. are more likely detached processes, true TNTs can form between brain types. Kempf et al. (2024) hypothesized that some of the observed bridges might connect pericytes to endothelial cells rather than other pericytes. In diabetic mouse retina, the authors observed an increased number in bridging structures but did not attributed this response to pericyte detachment and migration (as others had; Corliss et al., 2020; Pfister et al., 2008). *In vitro* experiments showed however that pericytes could emit TNTs toward stressed endothelial cells, transferring mitochondria, and restoring metabolic activity (Kempf et al., 2024). It is however unclear whether the structure presented on tissue sections are genuine TNT and not pericytes processes.

### 2.4 The non-pericyte candidates for bridging cells

In their 2012 study, Mendes-Jorge et al. (2012) investigated the morphology and marker expression of bridging cells in the mouse retina, uncovering several intriguing features. They found that these cells express markers shared by both endothelial cells and pericytes, such as CD34, poly-N-lactosamine, and CD13, while lacking expression of CD31, a key marker of endothelial cells, and actin. However, the limited co-staining combinations presented in this study do not allow for a clear determination of whether these markers are specific to bridging cells or are expressed separately in distinct cell types. Interestingly, the presence of condensed chromatin (indicative of a transcriptionally inactive state) and a higher prevalence of bridging cells in young animals evoke similarities with mesenchymal progenitor cells (Mendes-Jorge et al., 2012).

Telocytes, formerly known as interstitial Cajal-like cells, also share striking structural similarities with bridging cells (Popescu and Faussone-Pellegrini, 2010; Zhang et al., 2023). Present in the interstitial tissue of multiple organs, they extend long, thin processes, spanning hundreds of micrometers, from a small soma. However, the novelty of telocytes has been heavily debated, given their similarities with fibroblasts. Their lack of specific association with capillaries and their often convoluted, branching morphology make them unlikely candidates for the bridging cells observed in the CNS (Varga et al., 2019).

Fibroblasts, on the other hand, can play stabilizing roles in blood vessels and appear earlier than SMCs or pericytes during development. They help lay down basement membranes that guide new vessel growth (Rajan et al., 2020). It is interesting to note that nearly all reports of bridging cells describe their association with vascular basement membrane (Alarcon-Martinez et al., 2022, 2020; Ando et al., 1999; Cammermeyer, 1965, 1960; Corliss et al., 2020; Hou et al., 2018; Kempf et al., 2024; Leibnitz and Bär, 1988; Mendes-Jorge et al., 2012; Guseo and Gallyas, 1974; Pfister et al., 2008), an intricate relationship also shared by fibroblasts.

### 2.5 The lead of regressing vessels

Long, thin strands of conjunctive tissue extending between capillaries are not unfamiliar to neuroanatomists. Reports date back to the 19th century, with prominent figures such as Henle and Ramón y Cajal describing such structures in the nervous systems of various vertebrates, including humans (Cammermeyer, 1960). In a comprehensive 2010 review, Brown detailed the morphology and dynamics of “string vessels” (Brown, 2010), empty tubes of basement membrane resulting from vascular regression. These structures are frequently found in the aging brain, more prominently in Alzheimer’s disease, but also in the developing CNS, where they likely reflect the intense vascular remodeling of early life (Coelho−Santos and Shih, 2020).

In a 2025, Gao et al. (2025) extensively mapped regressing vessels across the mouse brain. They found that most of these strands contained components, and to a lesser extent, endothelial elements, embedded within basement membrane tubes. In fewer than 20% of cases, they observed bulging somas reminiscent of Cammermeyer’s delta cells, while fully embedded bipolar cells were seen in only ∼2% of string vessels. In human brains, these structures were slightly more frequent and appeared longer (Gao et al., 2025). Following experimental ischemia in mice, the number of regressing vessels increased and correlated with neuronal dysfunction, suggesting a potential link between vascular regression and impaired brain function (Gao et al., 2025).

## 3 Discussion

**FIGURE 2:**
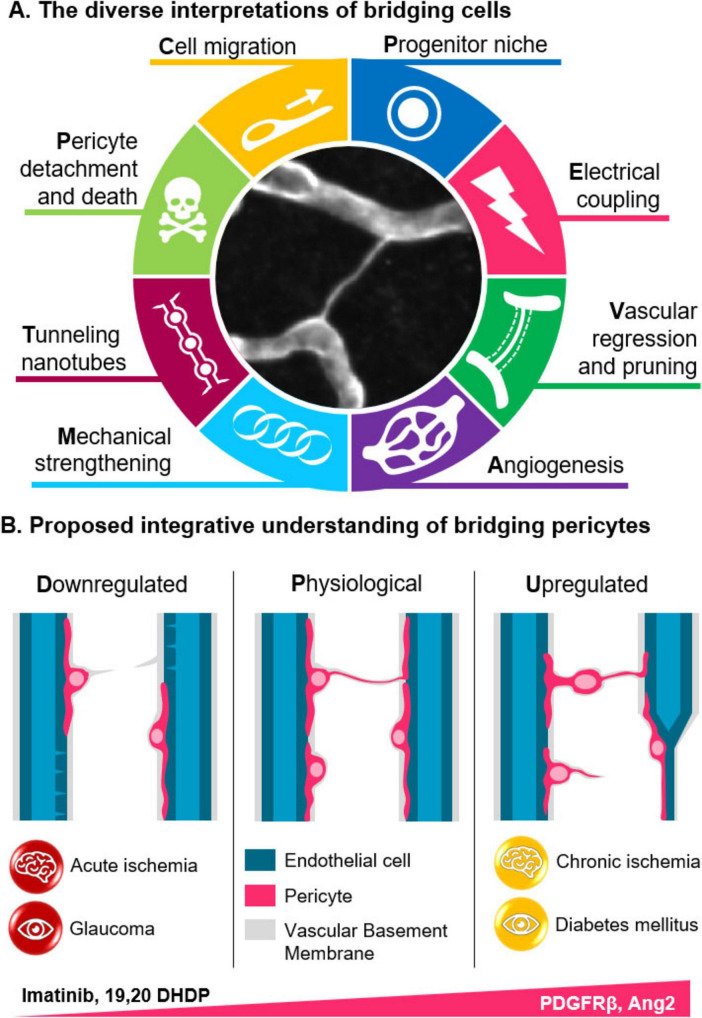
Significance and roles of bridging cells. **(A)** Bridging cells have been proposed to serve various roles, including functioning as progenitor cells, remnants of pruned capillaries, guides for angiogenesis, structural components, tunneling nanotubes involved in neurovascular coupling, or as migrating and detaching pericytes. **(B)** While bridging cells are presentr in healthy brains, their numbers increase significantly when pericyte migration is enhanced, such as in diabetes or following vascular regression after ischemia. In contrast, there bridges are disrupted in the acute phase of ischemia and in glaucoma. (PDGFRβ: Platelet-derived growth factor beta; 19,20-DHDP: 19,20-dihydroxydocosapentaenoic acid).

The varying descriptions (Table 1) and proposed functional roles of bridging cells and intercapillary strands are reminiscent of the historical ambiguities surrounding the definition of pericytes. In the absence of clear definitions, structures that appear morphologically identical (Figure 1C) can be subject to widely divergent interpretations. Indeed, bridging cells have been interpreted as signs of vascular regression (Gao et al., 2025; Guseo and Gallyas, 1974; Zimmermann, 1923), elements structuring capillary loops (Ando et al., 1999; Cammermeyer, 1960; Mendes-Jorge et al., 2012; Williamson et al., 1980), sites of angiogenesis (Leibnitz and Bär, 1988; Guseo and Gallyas, 1974), progenitor niches (Mendes-Jorge et al., 2012), indicators of pericyte migration or detachment (Corliss et al., 2020; Gao et al., 2025; Hou et al., 2018; Kempf et al., 2024; Leibnitz and Bär, 1988; Pfister et al., 2008), or even tunneling nanotubes (Alarcon-Martinez et al., 2022, 2020; Cammermeyer, 1965; Figure 2A). These interpretations are not mutually exclusive if we consider them as part of the same framework.

**TABLE 1:** Observations and interpretations of bridging cells in the central nervous system.

Reference	Context	Methods	Description of bridging cells	Interpretation (Figure 2A)
Zimmermann, 1923	Heart, brain and other tissues in reptiles, fishes, birds and mammals.	Brightfield microscopy on silver-stained sections	Processes of pericytes bridging capillaries.	Vascular regression
Cammermeyer, 1960	Brain and spinal chord in birds and mammals	Brightfield microscopy on PAS/gallocyanin-chrome alum stained sections	Delta cells (triangular nuclei) and intervascular strands. Some nuclei were located midway. Bridges number widely vary between species, with highest numbers in birds and smallest numbers in small mammals.	Mechanical stabilization
Cammermeyer, 1965	Brain and facial nucleus, cat and rabbit	Brightfield microscopy on PAS/gallocyanin-chrome alum stained sections	Delta cells (triangular nuclei) and Intervascular strands. Strands may contain several PAS-stained granules, while vacuoles occur near the nuclei of delta cells.	Transmission route
Guseo and Gallyas, 1974	Brain, human	Brightfield microscopy on silver-stained sections	Rare occurrences in adult humans but frequent in newborns. Propose 3 classifications depending on endothelial or pericytic origin.	Vascular regression Angiogenesis
Williamson et al., 1980	Muscle and retina, human	Fluorescence microscopy, basement membrane staining	Pericytes processes bridging capillaries. Pericytes are more tightly attached to vessels in retina than in muscle, bridges appear as pericyte processes.	Mechanical stabilization
Leibnitz and Bär, 1988	Brain, rhesus monkey, cat, rat, mice and shrew	Brightfield microscopy on silver-stained sections	Bridging cells are small, spindle of bell-shaped. Cells located midway of a strand (bipolar cells) have one thicker process attached to a capillary. Bridges share the basal lamina of attached vessels.	Pericyte migration Pericyte detachment and death Angiogenesis
Ando et al., 1999	Cochlea, gerbil and rat	Fluorescence microscopy with basement membrane and actin stainings, Electron microscopy	Pericyte-like cells connecting blood capillaries. Bridges are laminin-positive and the basal lamina is contiguous with the vessel. No filamentous actin observed in bridges.	Mechanical stabilization Route for transmission
Pfister et al., 2008	Retina of spontaneously diabetic Ins2Akita XLacZ mice	Brightfield microscopy with LacZ reporter in pericytes and SMCs.	Delta and bipolar cells assumed to be migrating pericytes. 117 migrating cells per mm2 of capillary area in controls vs 198 in diabetic mice.	Pericyte migration Pericyte detachment and death
Mendes-Jorge et al., 2012	Retina, human and glaucoma mouse model (methylcellulose injection)	Brightfield microscopy Fluorescence microscopy with basement membrane, endothelial and pericyte stainings	Intervascular bridging cells in vascular basement membrane, connexin-43 expression at junction with vessel. Expression of endothelial and pericyte stainings suggest different subtypes or endothelial-pericyte transitional types. Higher number of bridges in 2 months vs. 6 months mice.	Mechanical stabilization Progenitor niche
Hou et al., 2018	Cochlea, mice exposed to loud sounds	Fluorescence microscopy Biochemistry Electron microscopy	Detached and migrated pericytes, NG2 + and PDGFRb + . PDGFRb is upregulated in migrated pericytes. 1% of migrated pericytes in controls, 20 times more in loud sound-exposed mice, partially rescued by Imatininb.	Pericyte migration Pericyte detachment and death
Gao et al., 2025 (preprint)	Brain, human and mouse ischemia model (MCA occlusion)	2P *in vivo* microscopy Fluorescence microscopy on cleared tissues, basement membrane, pericyte and endothelial stainings Metabolomics	Pericytes stuck along regressing vessels, covered by basement membrane and astrocyte endfeet. In mouse 80% of bridges do not show associated somas, 17.8% have one attached soma (delta cell), 2.4% have a soma midway (bipolar cell). 262 bridges/mm3 in 3 months vs. 178 bridges/mm3 in 24 months mouse brains.	Vascular regression Pericyte detachment and death
Alarcon-Martinez et al., 2020	Retina, mouse eye ischemia (optic nerve ligature)	2P *in vivo* microscopy, with NG2 fluorescent reporter Calcium imaging Fluorescence microscopy Electron microscopy	Bridging cells (delta cells), bridges are interpreted as TNTs. Bridges are 0.5 um thick and contain F-actin and mitochondria. 51 bridges/mm2 in healthy mouse retina, corresponding to 28% of retinal pericytes.	Route for transmission Electrical coupling
Corliss et al., 2020	Retina, human and diabetes mouse model (streptozotocin)	2P *in vivo* microscopy Fluorescence microscopy with pericyte, endothelial and basement membrane stainings.	Basement membrane bridges are CD31-, but 20% are NG2 + . PDGF-BB injection increases NG2 + fraction to 30%. 17 bridges per mm of capillary in healthy mouse retina.	Pericyte migration Pericyte detachment and death
Alarcon-Martinez et al., 2022	Retina, mouse glaucoma (magnetic beads injection)	2P *in vivo* microscopy Calcium imaging	Bridges are interpreted as inter-pericyte TNTs, 25% of which are broken in glaucoma.	Route for transmission Electrical coupling
Kempf et al., 2024	Pericytes/endothelial cells co-cultures from humans; Retina, mouse model of diabetes (Ins2Akita)	Fluorescence microscopy with pericyte, endothelial and TNT markers Proteomics Metabolomics	Pericytes TNTs to endothelial cells, migrating pericytes. 2 TNTs per field of view in wild-type mice vs 5 TNTs per field of view in diabetic mice.	Route for transmission

PAS, periodic acid-schiff; PDGFRb, platelet-derived growth factor β; MCA, middle cerebral artery; TNT, tunneling nanotubes.

First, while some authors have identified bridges as TNTs (Alarcon-Martinez et al., 2022, 2020), these structures do not appear to differ fundamentally from conventional cellular processes. Rather than connecting pericytes to each other, they maintain cellular continuity by linking a single pericyte’s extensions across neighboring capillaries. The observation of electrical coupling between pericytes separated by these bridges is not unexpected, given the well-established intimate coupling between pericytes and endothelial cells (Kovacs-Oller et al., 2020). It is therefore more plausible that the observed pericyte-pericyte coupling is indirectly mediated via endothelial cells, rather than through a direct TNT-like structure. Regarding the functional significance of these bridges in neurovascular coupling, it remains to be demonstrated whether they play an active role or simply represent incidental anatomical arrangement. As suggested by Leibnitz and Bär (1988), the low frequency of these bridges makes it unlikely that they contribute substantially to neurovascular dynamics.

The presence of *bona fide* TNTs within connective strands remains conceivable, although they are typically described as lacking association with basement membranes (Zhang, 2011). While Kempf et al. (2024) demonstrated that pericytes can transfer mitochondria to damaged endothelial cells via TNTs, yet the relevance of intercapillary connections remains puzzling, as most endothelial cells are already covered by pericytes, offering a more immediate opportunity for support.

The hypothesis for pericyte migration is better established and supported by different studies (Corliss et al., 2020; Hou et al., 2018; Pfister et al., 2008). Angiotensin-2 and PDGFRβ upregulation appear to increase pericyte motility and detachment, potentially resulting in the presence of bipolar cells transiting across these bridges. The fate of detached pericytes remains to be determined; however, rather than undergoing cell death, some may transdifferentiate into other cell types such as microglia (Sakuma et al., 2016; Nirwane and Yao, 2022; Morris et al., 2023) or myofibroblasts (Zhao et al., 2022). Interestingly, migrating pericytes sometimes extend processes toward other vessels, a behavior that contrasts with the typical detachment and migration patterns observed during development (Payne et al., 2021). It is conceivable that some pericytes migrate within the basement membrane, only to become irreversibly trapped within the fibrotic remnants of regressed capillaries, where they may ultimately die.

Vascular regression and pruning are well known sources of connective strands in the CNS (Brown, 2010; Ouarné et al., 2021; Reissenweber and Pessacq, 1971). As endothelial cells retract, they can leave behind pericytes encased within empty sleeves of basement membrane, structures that likely give rise to bridging cells (Gao et al., 2025; Ouarné et al., 2021). Structural features support this mechanism: for example, the bending of capillaries near the origin of a bridge may reflect a residual angle from a former bifurcation rather than mechanical tension (Leibnitz and Bär, 1988). Rare occurrences of Y- or V-shaped bridges can be explained by the pruning of several vessels. Future studies could help identify bridges resulting specifically from vascular regression by detecting endothelial-derived components of the basement membrane. However, the factors determining pericyte fate during vascular regression remain unclear. Notably, Gao et al. (2025) claim that bridging cells can persist for months after endothelium loss.

Several factors have been reported to influence the number of bridging cells. In the retina, hypometabolic conditions such as glaucoma or acute ischemia are associated with a reduction in bridges numbers (Alarcon-Martinez et al., 2022, 2020), whereas hypermetabolic states like diabetes promote pericyte migration and an increase in bridges formation (Corliss et al., 2020; Pfister et al., 2008). This effect can be counteracted by the PDGFRβ inhibitor imatinib (Hou et al., 2018; Figure 2B). Although observations in the brain remain limited, they support the idea that vascular regression is a major source of bridging structure, as seen during capillary pruning in development (Brown, 2010; Leibnitz and Bär, 1988) and after ischemic events (Gao et al., 2025). Moreover, substances that disrupt plasma membrane dynamics and the formation of TNTs, such as 19,20-dihydroxydocosapentaenoic acid (19,20-DHDP), also reduce bridge numbers (Kempf et al., 2024) – albeit through a distinct mechanism.

Intriguingly, some bridging cells express markers indicative of endothelial-pericyte hybrids or even progenitor cells (Mendes-Jorge et al., 2012). The involvement of pericytes in vascular growth is well established (von Tell et al., 2006; Payne et al., 2021), and endothelial cells are known to proliferate within empty basement membrane sleeves (Archer et al., 1991). Bridging cells have been proposed to serve as guides for angiogenesis (Leibnitz and Bär, 1988), though this mechanism differs from the classical role of endothelial tip cells in forming new vascular connections (Payne et al., 2021). Rather than promoting regrowth, pericytes may act as stabilizers, potentially inhibiting endothelial regeneration after pruning (Gao et al., 2025). While this function is essential during development, it could hinder vascular repair in the aging brain. Such antagonistic pleiotropic mechanisms are not unprecedented. Targeting bridging cells, while preserving the basement membrane sleeve, might offer a strategy to promote endothelial regrowth. It is also worth noting that the condensed chromatin observed in bridging cells (Mendes-Jorge et al., 2012) may be a sign of cellular senescence.

Far from being mere anatomical curiosities, bridging cells and connective strands raise important questions about cerebrovascular dynamics. While pericyte loss has been reported across a wide range of conditions, including Alzheimer’s (Ding et al., 2020), stroke (Hall et al., 2014), and even healthy aging (Soto et al., 2015), the mechanisms underlying pericyte detachment remain poorly understood. Studies such as those by Corliss et al. (2020) and Hou et al. (2018) suggest that bridging pericytes may arise from dysregulated cell migration and recruitment; however, how these processes ultimately lead to pericyte death remains unclear.

Conversely, the recovery or replacement of pericytes following injury has been scarcely documented. It is plausible that such recovery would require cells to migrate along the vascular tree, potentially leading to the formation of new bridging structures. Interpreting the presence of bridging cells as indicators of vascular remodeling, whether regenerative or degenerative, is compelling, particularly given that similar structures may emerge during vascular pruning (Brown, 2010). Assessing whether such remodeling originates from pericytes or endothelial cells might be addressed by further investigation of the matrix components within these bridges.

Bridging pericytes may also play an active role in the pruning process by stabilizing endothelial cells and thereby inhibiting vascular regrowth (von Tell et al., 2006). While this stabilizing function facilitates pruning during development, it may conversely hinder vascular recovery in aging or disease, making these cells a potential therapeutic target for restoring brain perfusion in conditions of hypoperfusion.

Interestingly, some researchers suggest that the bridging cells may serve functions beyond being transient by-products of vascular remodeling. Although observations of the temporal dynamics of these structures are limited, available evidence suggests that a significant proportion of bridges persist for months (Gao et al., 2025). This longevity raises the possibility of functional roles for bridging cells, an idea beginning to be explored in dedicated studies (Alarcon-Martinez et al., 2020; Kempf et al., 2024). Nevertheless, *in vivo* functional investigations remain scarce and are currently limited to the retina, which is more accessible than other regions of the CNS

## 4 Conclusion

Since their discovery alongside pericytes, bridging cells have been examined from a wide range of perspectives. Despite the diverse interpretations they have attracted, these elusive cells are now generally recognized as pericytes whose processes extend between capillaries. As research has progressed from descriptive studies to functional analyzes, new hypotheses have emerged regarding their origin and relevance within the central nervous system. Although pericyte migration and vascular regression are considered the two primary sources of these capillary bridges, the underlying mechanisms remain to be fully elucidated. Unraveling these pathways holds promises for the identification of therapeutic targets in vascular diseases of the CNS and beyond.
